# Stiffness Separation Method for Damage Identification in Continuous Rigid Frame Bridges

**DOI:** 10.3390/s25237141

**Published:** 2025-11-22

**Authors:** Feng Xiao, Linger Xu, Yu Yan, Yujiang Xiang

**Affiliations:** 1 School of Safety Science and Engineering, Nanjing University of Science and Technology, Nanjing 210094, China; xulinger@njust.edu.cn (L.X.); yy915160352@njust.edu.cn (Y.Y.); 2 Mechanical and Aerospace Engineering, Oklahoma State University, Stillwater, OK 74078, USA; yujiang.xiang@okstate.edu

**Keywords:** CRF bridges, stiffness separation method, damage identification, substructure, large-scale structures

## Abstract

Optimization-based damage identification in continuous rigid frame (CRF) bridges faces challenges. As the degrees of freedom of the structure increase, the complexity of the objective functions increases significantly, making convergence more difficult to achieve. This study introduces a stiffness separation method for damage identification in CRF bridges. This method decomposes the overall stiffness matrix of the bridge into the required stiffness submatrices, which greatly simplifies the objective function for identifying structural damage. By dividing the stiffness matrix into smaller stiffness submatrices, the proposed method reduces computational complexity and improves damage detection efficiency. Two CRF bridges are presented to verify the effectiveness of the proposed method.

## 1. Introduction

Bridges, as key infrastructure components, are crucial for both transportation and economic development. For long-span bridges, continuous rigid frame (CRF) bridges are favored because of their structural stability, continuity, straightforward construction, aesthetics, and broad application prospects [1,2,3,4]. However, bridges face various challenges. Factors such as traffic loads, corrosion, natural disasters, etc., can cause damage to bridges [5,6,7,8], resulting in a reduction in the stiffness of their elements. Stiffness is a crucial metric for assessing the condition of CRF bridges. A reduction in stiffness usually triggers a change in the bridge characteristics [9,10,11,12]. Therefore, the application of advanced methods to detect damage and ensure structural safety is essential [13,14,15].

The behavior of bridge structures can be categorized Into two main response characteristics: static and dynamic [16]. Static response characteristics are mainly influenced by changes in static performance parameters such as strain, static displacement, deflection, structural stiffness, and material properties. Researchers have studied structural damage related to static responses. Sanayei et al. [17] conducted static vehicle loading tests during the construction phase by obtaining strain measurements from bridge instrumentation. Strain data from nondestructive testing and parameters such as cross-sectional area were used to calibrate a baseline finite element model of elasticity. Further, Sanayei et al. [18] introduced a new method that employs multi-response estimation for simultaneously determining the stiffness parameters in finite element models. Banan and Hjelmstad [19,20] used measured displacements subjected to static force to develop two algorithms for measuring member properties obtained from finite element models. Through simulation experiments, they investigated the performance of the estimators to assess the influence of force and displacement factors on the performance of the algorithms. Further, Hjelmstad and Shin [21] proposed an optimized algorithm for recognizing damage, even in the presence of noise and limited data. Fernandez-Navamuel et al. [22] used a deep autoencoder neural network to analyze the dynamic responses of bridges under different damage scenarios. Even in the presence of measurement errors and environmental changes, they were able to make more accurate damage predictions through bridge experiments on the Infante Dom Henrique and Z24 Bridges. Le et al. [23] explored methods for identifying and quantifying the damage to Euler–Bernoulli beams due to variations in static deflection. In summary, first, damage manifests fundamentally as a reduction in structural stiffness. Static responses are linked to the structural stiffness matrix. Therefore, changes in static measurements are a direct indicator of stiffness degradation and offer insight into the extent of the damage. Second, localized damage often induces pronounced changes in local strain distribution. Static damage indicators are more sensitive to localized damage. Third, strain and displacement sensors are widely used in bridge monitoring, enabling direct utilization of installed static sensors for bridge damage identification.

To assess the damage to CRF bridges, Wu et al. [24] proposed a method for updating finite element models that combines dynamic and static long-measurement strain responses to enhance its ability to make accurate predictions by optimizing both global and local structural parameters such as bending stiffness. Cheng and Liao [25] used a revised finite element model to predict higher-order modal frequency measurement changes and validated these predictions using a concrete rigid frame continuous highway bridge. Zhang et al. [26] implemented a model updating approach that relies on the properties of free-wave behavior in a 23-span CRF bridge project by minimizing the disparity between the computed free-wave properties and those detected. Deng et al. [27] devised a method based on wavelet packet norm entropy for identifying seismic damage to curved CRF bridges. This method monitors the dynamic response of a bridge subjected to seismic stimulation. The dynamic responses in different directions are then compared to determine the most sensitive damage indices. Niu et al. [28] proposes a bridge damage identification method by combining finite element model updating and a modal strain energy-based index. Wang et al. [29] exploited coded images and convolutional neural networks to enhance bridge damage detection for safety and validated the proposed method using a 5-span CRF bridge girder model.

Although studies on damage identification have been conducted on CRF bridges with few spans, challenges arise for large and complex bridges. Table 1 presents a compilation of existing operational CRF bridges worldwide. Large and complex CRF bridge structures face several challenges. First, as the degrees of freedom (DOF) of a structure increase, the computational requirements also increase. Second, as the damage indicators increase, the objective function becomes increasingly difficult to converge.

The stiffness separation method [30,31,32,33] is more suitable for dealing with large-scale structures. To obtain a specified solution, each substructure is analyzed independently. The stiffness separation method has three main advantages. First, the global structure is replaced by more feasible reduced substructures because the analysis of small-scale system matrices is easier and faster. Second, the substructures are evaluated independently. This feature has broad application prospects for damage identification and model updating. For example, if uncertainty exists or if the damage site occurs locally in the structure, only the substructure containing the damage site is reanalyzed. The stiffness separation method reduces computational complexity and decreases the number of iterations in the optimization procedure.

This study uses the stiffness separation method to identify damages to CRF bridges. To accomplish this, substructures are isolated from the entire structure. An objective function is then established by comparing the analytical displacements of the substructure with the corresponding measured values and is optimized to identify damage. Thus, the proposed method can substantially decrease the number of parameters to be identified, simplify the objective equation. The effectiveness of this approach is validated on both four-span and twelve-span CRF bridges.

## 2. Stiffness Separation Method

The stiffness separation method precludes an overall modeling solution for large and complex structures, thereby significantly enhancing the efficiency of damage identification. Damage identification techniques based on the stiffness separation method utilize additional sensors to isolate a partial model from the overall structure. Equations are then formulated based on the structural characteristics of the partial model. The primary concept is that displacement sensors are used to isolate the substructure from the overall structure. The relevant components of the substructure, including the matrices and vectors, are separated from the overall stiffness equations. This process enables equilibrium equations to be established for the substructure. Finally, the unknown displacements within the target region of the substructure are computed, and the following equilibrium equations for the substructure are thus formulated.

(1) $$K_{m\;\times \;n}D_{n}=Q_{m}\;$$

where $K_{m\;\times \;n}$ is a submatrix of the stiffness matrix of the substructure, $D_{n}$ is the displacement vector of the substructure containing the displacements of all DOFs of the substructure, and $Q_{m}$ is the load vector of the substructure including the DOFs of the unknown displacements of the substructure. The subscripts denote the size of the matrices or vectors: m is the number of unknown displacements in the substructure and n is the total number of DOFs in the substructure. For computational convenience, Equation (1) can be derived as follows:

(2) $$\left[\begin{array}{cc} K_{1} & K_{2} \end{array}\right]\left[\begin{array}{c} D_{u}\\ D_{k} \end{array}\right]=\;Q_{m}\;$$

where $K_{1}$ is an *m* × m square matrix and $K_{2}$ is an m × (n – m) matrix. $D_{u}$ and $D_{k}$ are the unknown and known displacement vectors of the substructure, respectively. The compatibility between the stiffness submatrices and displacements should be considered during matrix partitioning.

Structural damage often leads to a reduction in stiffness. Therefore, a decrease in the elastic modulus of members can be an indication that a member has been damaged [25,34,35]. In this study, a change in the elastic modulus is used as an indicator of damage. When damage exists in an isolated substructure, the stiffness submatrix constructed from the substructure contains parameters because the elastic modulus of the damaged components is unknown. The unknown parameters are denoted as $E_{d}$. Therefore, the analyzed value of the unknown displacements in the substructure can be obtained using Equation (3).

(3) $$D_{u}={\;K}_{1}^{-1}\left(Q_{m}-K_{2}D_{k}\right)\;$$

In Equation (3), $D_{u}$ contain the unknown parameter $E_{d}$. Then, parameter estimation is performed to determine the unknown parameters based on the measured and analytical responses of the structure. For this study, an objective function is established as shown in Equation (4).

(4) $$f=\sum_{i=1}^{M}{\left[D_{i}^{\prime }-D_{i}\right]}^{2}$$

where $D_{i}^{\prime }$ and $D_{i}\;$are the i-th measured and analytical displacement, and *M* is the total number of measurement points. When the difference between the analytical and measured values of the displacements is extremely small, the objective function tends toward 0. The objective function is optimally solved to obtain the parameter values when the minimum value is achieved.

(5) $$E_{d}^{*}=\arg\;\underset{E_{d}}{\min}\left(\;f\;\right)$$

where $E_{d}^{*}$ is the parameter values after the optimization iteration is terminated.

## 3. Damage Identification in CRF Bridges

### 3.1. Four-Span CRF Bridge

**Figure 1:**
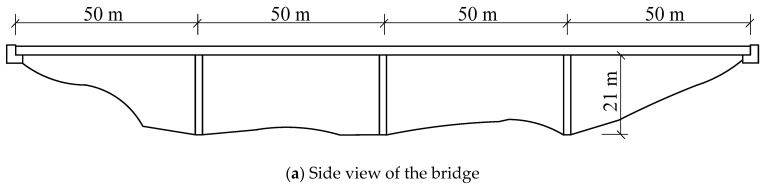

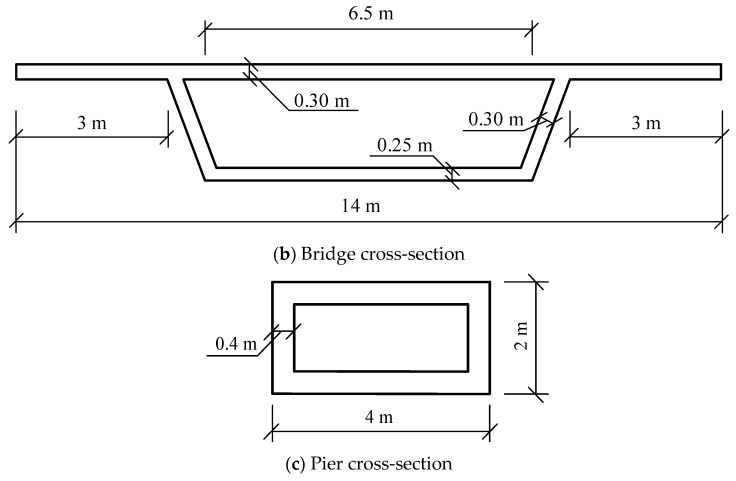
Schematic of the four-span CRF bridge. (**a**) Side view of the bridge. (**b**) Bridge cross-section. (**c**) Pier cross-section

**Figure 2:**
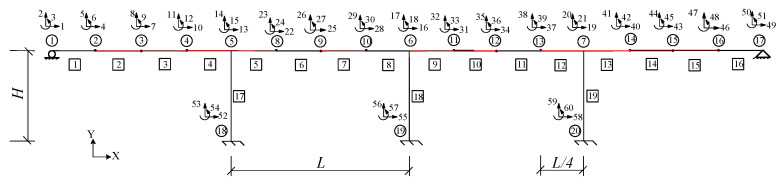
Structural diagram.

The CRF bridge comprised four 50 m long spans for a total length of 200 m, with a pier height of 21 m and a hollowed-out rectangular shape in cross-section. The bridge structure and related cross-section diagrams [36,37] are shown in Figure 1. The elastic modulus *E* of the bridge is 20.6 Gpa. The deck has a cross-sectional area of 8.447 m^2^ and moment of inertia of 6.519 m^4^. A structural diagram of the bridge model is shown in Figure 2, where *H* and *L* are 21 and 50 m, respectively. Red indicates the location of the damage to the structure. The bridge model is established based on the force-displacement stiffness relationship. The boundary conditions are shown in Figure 2.

To simplify the damage identification investigations on an entire bridge structure, the stiffness separation method divides the entire structure into several substructures, thus transforming the damage identification problem of the entire structure into one of several substructures, where the substructures can be analyzed rather than the entire structure. This reduces the size of the matrix, decreases the quantity of unidentified parameters included in the operation, and reduces the computational difficulty, thereby diminishing the iterations necessary to achieve the objective function. The number of iterations necessary to attain the optimal value of the objective function is thus reduced and computational efficiency is improved.

**Figure 3:**
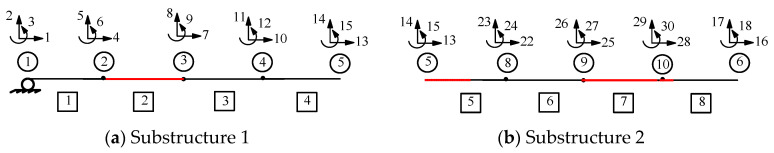

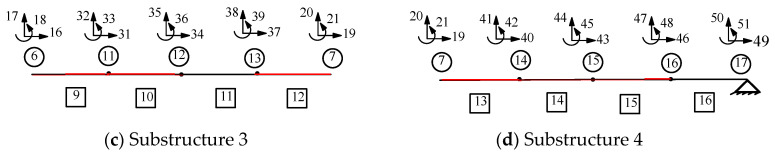
Schematic of the bridge substructure for the four-span CRF bridge. (**a**) Substructure 1 (**b**) Substructure 2 (**c**) Substructure 3 (**d**) Substructure 4.

Therefore, the stiffness separation method can be used to determine the extent of damage to a CRF bridge divided into four substructures. Figure 3 shows a schematic of the four substructures separated by nodes 5, 6, and 7. Table 2 lists the extent of the damage to the structure.

An independent load is applied to each substructure. In this study, the measured displacements are derived from artificially induced damage scenarios. For substructure 1, a vertical downward load with a magnitude of 100 kN is applied at a DOF of 8. Displacements under 13, 14, and 15 DOFs are measured as boundary conditions for substructure 1. An objective function for substructure 1 is defined based on its measured and analytical displacements under 5, 8, and 11 DOFs. Similarly, for substructure 2, a vertical downward static load of 100 kN is applied at a DOF of 26. The measured responses under 13, 14, 15, 16, 17, and 18 DOFs are separated from substructure 2. An objective function for substructure 2 is defined based on its measured and analytical displacements under 23, 26, and 29 DOFs. For substructure 3, a vertical downward static load of 100 kN is applied at a DOF of 35. The responses under 16, 17, 18, 19, 20, and 21 DOFs are measured for separation. An objective function for substructure 3 is established based on its measured and analytical displacements under 32, 35, and 38 DOFs. For substructure 4, a vertical downward load of 100 kN is applied at a DOF of 44. The measured responses under 19, 20, and 21 DOFs are used to separate substructure 4. An objective function for substructure 4 is defined based on its measured and analytical displacements under 41, 44, and 47 DOFs.

**Figure 4:**
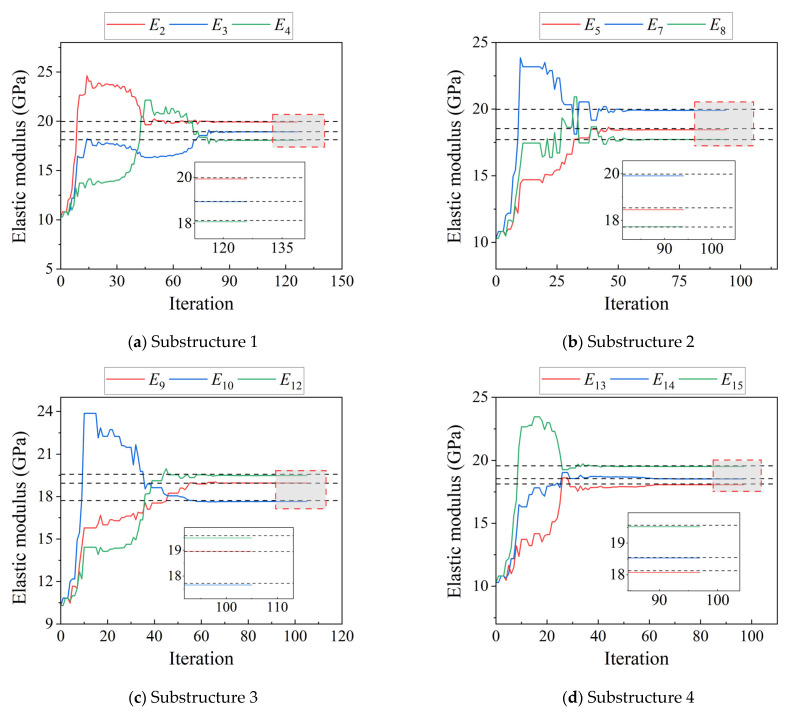
Iterative plot of the substructure parameters for a four-span CRF bridge with a 0.2% error. (**a**) Substructure 1 (**b**) Substructure 2 (**c**) Substructure 3 (**d**) Substructure 4.

In this study, the effect of measurement errors on damage identification is investigated, including levels of 0.2%, 0.5%, 1%, and 2%. First, a 0.2% error is added to each measured displacement. Damage identification is then performed on the separated substructures, and the objective function of each separated substructures is optimized using the Nelder–Mead simplex method [38,39]. The initial value of the elastic modulus is set to 10.3 GPa, which is half of the “as-built” value. The results of the parameter iterations for substructures 1, 2, 3, and 4 are shown in Figure 4. The horizontal axis denotes the number of iterations of the objective function, and the vertical axis denotes the value of the elastic modulus. Dashed lines indicate the damage conditions for the identified parameters (Table 2).

Mean relative error (MRE) is a statistical metric that measures the discrepancy between the estimated and true values, that is, it represents the average percentage of the prediction error relative to the true value.

(6) $$\mathrm{MRE}=\frac{1}{N}\sum_{j=1}^{N}\frac{\left|E_{d_{j}}^{\prime }-E_{d_{j}}^{*}\right|}{E_{d_{j}}^{\prime }}\;$$

where N denotes the total number of damaged members. $E_{d_{j}}^{*}$ and $E_{d_{j}}^{\prime }$ denote the optimal and “as-is” state of the elastic modulus of the member, respectively.

**Figure 5:**
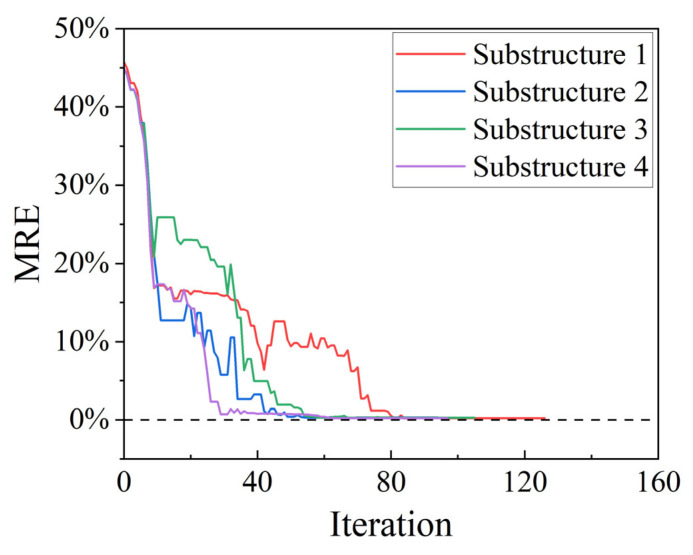
MRE trend for a 0.2% error.

Figure 5 shows the MRE curves for the iterative process with a 0.2% error. The four lines represent the change in MRE of substructures 1, 2, 3, and 4 as the iteration process progresses. The results show that the MRE values tend toward zero.

**Figure 6:**
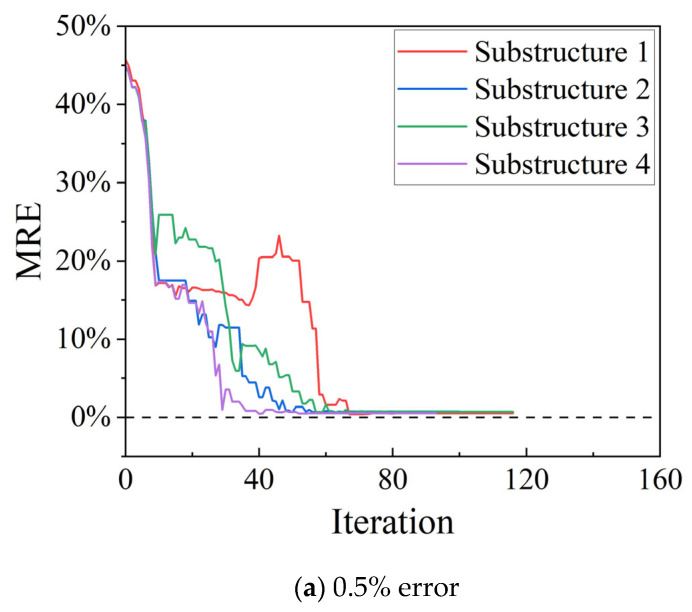

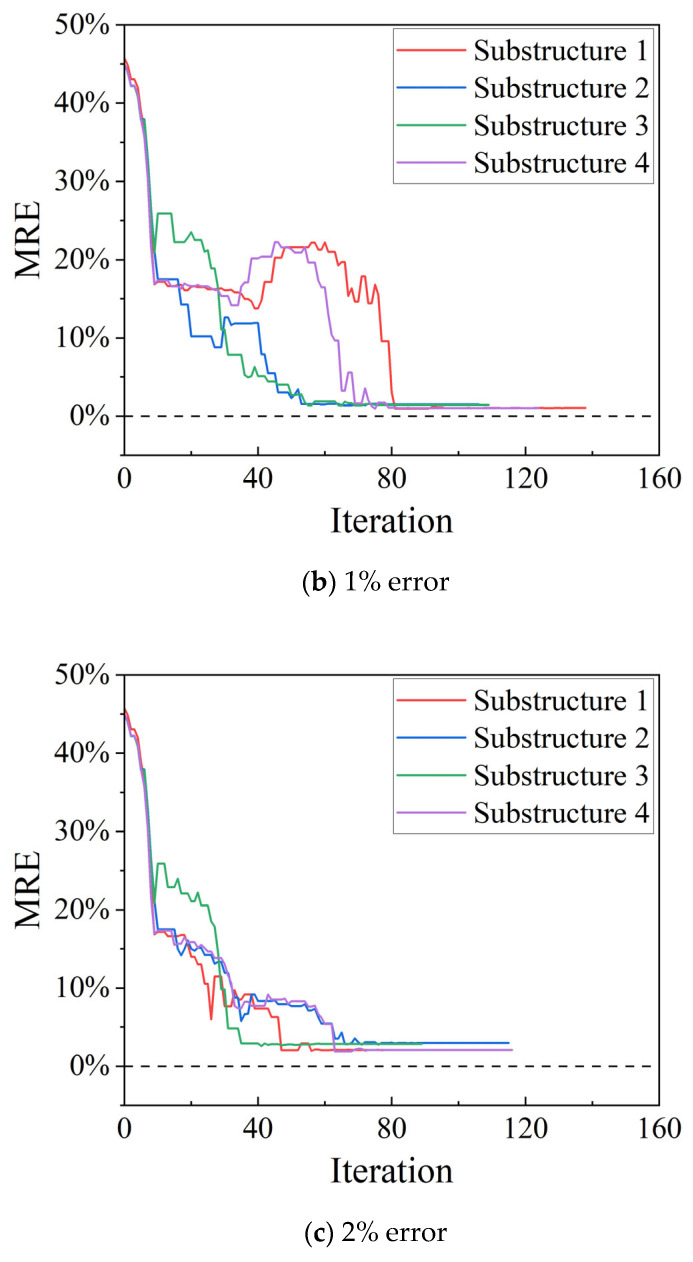
MRE trends for different measurement errors. (**a**) 0.5% error (**b**) 1% error (**c**) 2% error.

Then, displacement measurements with errors of 0.5%, 1%, and 2% are studied. Figure 6 shows the MRE iterative plots under different measurement errors. Structural damage identification in the four-span rigid bridge verifies the accuracy of the investigated structural stiffness separation method for rigid bridges, even in the presence of measurement errors.

### 3.2. Twelve-Span CRF Bridge

**Figure 7:**
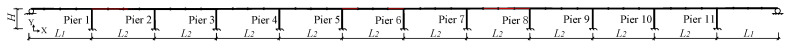
Schematic of the twelve-span CRF bridge.

**Figure 8:**
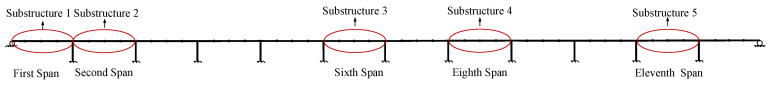
Schematic of substructure separation.

**Figure 9:**
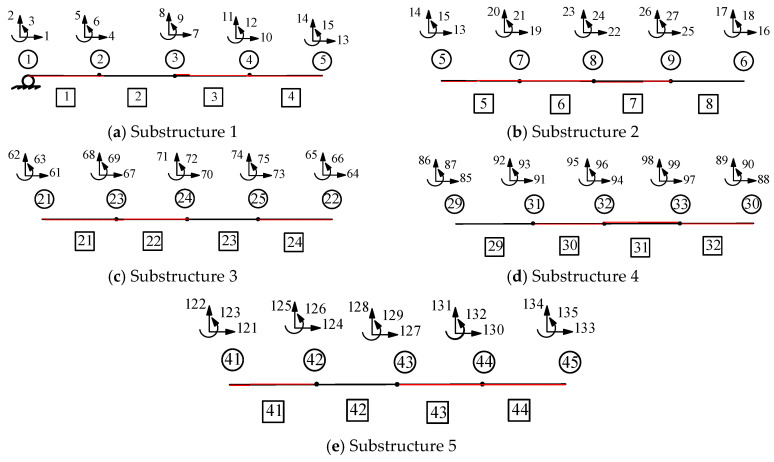
Schematic of the bridge substructure for the twelve-span CRF bridge. (**a**) Substructure 1 (**b**) Substructure 2 (**c**) Substructure 3 (**d**) Substructure 4 (**e**) Substructure 5.

This section applies the proposed method to a large-scale structure. The CRF bridge is a twelve-span bridge comprising two spans of 32.08 m and ten spans of 43.59 m, with 11.99-m-high piers. In Figure 7, $L_{1}$ is 32.08 m, $L_{2}$ is 43.59 m, and *H* is 11.99 m. The girders and piers have elastic modulus of 24.86 and 22.41 GPa, respectively. The cross-sectional area of the girders is 6.94 m^2^, and the moment of inertia is 3.39 m^4^. The cross-sectional area of the piers is 5.83 m^2^, and the moment of inertia is 0.72 m^4^. Figure 8 and Figure 9 show the locations of the damage and substructures. Table 3 lists the values for the damage conditions.

In Figure 9a, for substructure 1, a vertical downward load with a magnitude of 100 kN is applied at node 3. Displacements under 13, 14, and 15 DOFs are measured as boundary conditions for substructure 1. An objective function for substructure 1 is defined based on its measured and analytical displacements under 5, 8, and 11 DOFs. The initial elastic modulus for optimization is set to 12.43 GPa. Similarly, the objective functions of substructures 2, 3, 4, and 5 are established.

**Figure 10:**
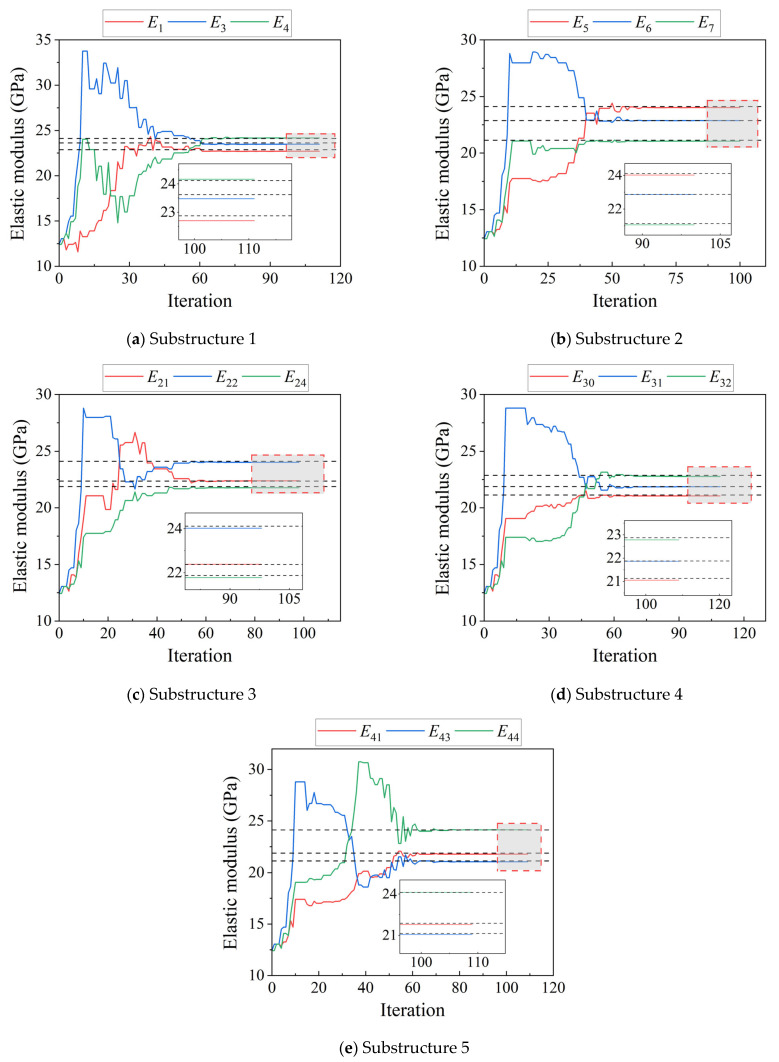
Iterative plots of the substructure parameters for the twelve-span CRF bridge with a 0.2% measurement error. (**a**) Substructure 1 (**b**) Substructure 2 (**c**) Substructure 3 (**d**) Substructure 4 (**e**) Substructure 5.

**Figure 11:**
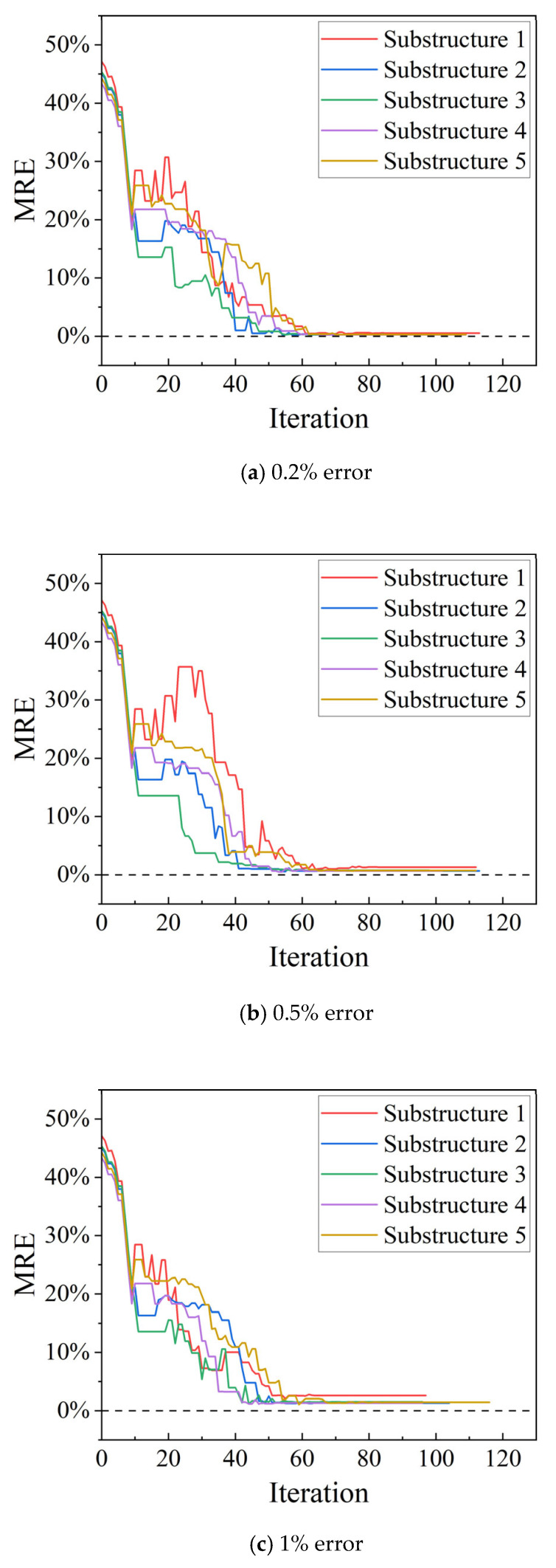

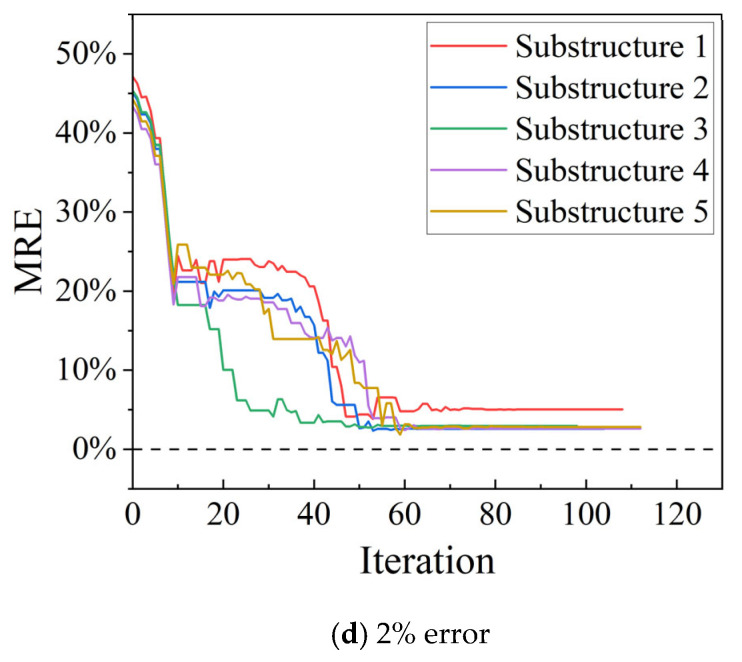
MRE results for the twelve-span bridge. (**a**) 0.2% error (**b**) 0.5% error (**c**) 1% error (**d**) 2% error.

Figure 10 shows the results of the parameter iterations for the four substructures at a 0.2% measurement error. Figure 11 shows the MRE iterative plots at 0.5%, 1%, and 2% measurement errors.

The accuracy of the identified results is then investigated under different error levels. Table 4 lists the final MREs of the two bridges. According to the results, in each case, the errors in the identified results are relatively small, and all values are less than 4%.

**Figure 12:**
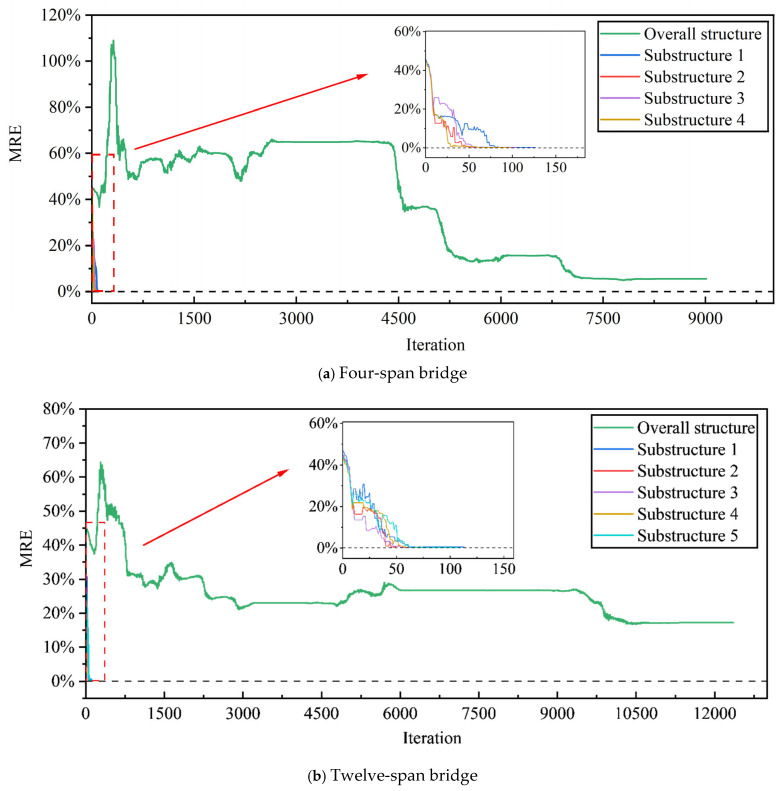
MRE trend at a 0.2% measurement error. (**a**) Four-span bridge (**b**) Twelve-span bridge.

Next, to study the efficiency of the stiffness separation-based damage identification, direct damage identification is investigated. In Figure 12, the lines of the overall structure show the 0.2% error MRE results for the four- and twelve-span bridges based on direct damage identification. The subgraphs show the corresponding stiffness separation-based damage identification results.

Based on the direct damage identification method, the number of iteration steps of the overall structure is 9015 (Figure 12a). Further, based on the stiffness separation method, the number of iteration steps of substructures 1, 2, 3, and 4 are 126, 94, 105, and 97, respectively. The final MRE value for direct damage identification is 5.49%, and the MRE value of the substructure tends toward zero.

As shown in Figure 12b, the final MRE value for direct damage identification is 17.19%, and the MRE value of the substructure tends toward zero. The number of iteration steps for the overall structure is 12,353, and the number of iteration steps for substructures 1, 2, 3, 4, and 5 are 113, 100, 98, 109, and 109, respectively. Overall, the stiffness separation method can effectively reduce the number of iterations and improve the efficiency of damage identification.

## 4. Conclusions

In this study, a stiffness separation method is used to investigate damage identification in four- and twelve-span CRF bridges. The overall structure is decomposed into different substructures and damage identification is performed separately on each substructure. By comparing the identification results from the overall structure and the substructures, the stiffness separation method is demonstrated to be an efficient and accurate approach for CRF bridge damage identification, effectively reducing the number of iterative steps of damage identification and improving computational efficiency. The advantage of the stiffness separation method is that by decomposing the overall structure into multiple substructures, the damage identification problem of the overall structure can be converted into a damage identification problem of multiple substructures. This effectively reduces the matrix size and the number of unknown parameters, thereby reducing computational complexity and improving identification efficiency.

## Figures and Tables

**Table 1:** Summary of the existing CRF bridges in operation.

Name	Length (m)	Spans	Country
Quanzhou Bay sea-crossing Bridge	4340	62	China
Confederation Bridge	11,080	45	Canada
Quhai rigid frame Bridge	768.6	29	China
US-24 Viaduct	378.9	17	USA
Río Nalón Viaduct	1100.85	17	Spain
Stana Clara Bridge	500.06	12	USA
Xilamulun River Bridge	1460	9	China
Wenming Bridge	852	8	China
Aigawa Bridge	636	8	Japan
Renyi River Bridge	740	6	China

**Table 2:** Damage to the structure of a four-span CRF bridge.

Substructure	Damage Parameters	Degree of Damage
1	$E_{2},E_{3},E_{4}$	3%, 8%, 12%
2	$E_{5},E_{7},E_{8}$	10%, 3%, 14%
3	$E_{9},E_{10},E_{12}$	8%, 14%, 5%
4	$E_{13},E_{14},E_{15}$	12%, 10%, 5%

**Table 3:** Damage to the twelve-span CRF bridge structure.

Damage Scenario	Damage Parameters	Degree of Damage
1	$E_{1},E_{3},E_{4}$	8%, 5%, 3%
2	$E_{5},E_{6},E_{7}$	3%, 8%, 15%
3	$E_{21},E_{22},E_{24}$	10%, 3%, 12%
4	$E_{30},E_{31},E_{32}$	15%, 12%, 8%
5	$E_{41},E_{43},E_{44}$	12%, 15%, 3%

**Table 4:** MRE mean values under different measurement errors.

Error Level	0.2%	0.5%	1%	2%
Four-span bridge	0.25%	0.63%	1.26%	2.48%
Twelve-span bridge	0.33%	0.82%	1.63%	3.20%

## Data Availability

Data is contained within the article.
